# Preparation of Styrene-Butadiene Rubber (SBR) Composite Incorporated with Collagen-Functionalized Graphene Oxide for Green Tire Application

**DOI:** 10.3390/gels8030161

**Published:** 2022-03-04

**Authors:** Anish Khan, Lau Kia Kian, Mohammad Jawaid, Aftab Aslam Parwaz Khan, Maha Moteb Alotaibi, Abdullah M. Asiri, Hadi M. Marwani

**Affiliations:** 1 Center of Excellence for Advanced Materials Research, King Abdulaziz University, Jeddah 21589, Saudi Arabia; draapk@gmail.com (A.A.P.K.); aasiri2@gmail.com (A.M.A.); hmarwani@kau.edu.sa (H.M.M.); 2 Chemistry Department, Faculty of Science, King Abdulaziz University, Jeddah 21589, Saudi Arabia; mmsalotaibi@kau.edu.sa; 3 Laboratory of Biocomposite Technology, Institute of Tropical Forestry and Forest Products (INTROP), University Putra Malaysia (UPM), Serdang 43400, Selangor, Malaysia; laukiakian@gmail.com

**Keywords:** SBR, collagen fibrils, tire, biodegradable, composite, automobile

## Abstract

Styrene-butadiene rubber (SBR) is a synthetic polymer primarily used in the tire industry, due to its good collaborative properties with additives and fillers. In the present work, we aim to synthesize an SBR composite reinforced with graphene oxide filler to be made biodegradable. In composite preparation, we fabricated styrene-butadiene rubber/graphene oxide/collagen (SBR/GO/COL) composites by adding a biodegradable biomolecule of elastin collagen fillers at 1.5 wt% and 2.5 wt%. Those prepared SBR/GO/COL composites, along with pure SBR and SBR/GO as control samples, were characterized using advanced analysis techniques, and their biodegradability was also evaluated. From microscopy examination results, the morphology of pure SBR had been improved after the addition of GO for SBR/GO composite by revealing a compact structure with a smoother surface. As for the SBR/GO/1.5COL sample, the 1.5 wt% COL filler was found to be effectively embedded in the SBR/GO matrix. However, the 2.5 wt% COL amount led to the formation of an aggregated structure in the SBR/GO/2.5COL sample due to the unreacted interface between COL filler and SBR/GO. The porosity had also been improved for SBR/GO/1.5COL sample, imparting it with a surface area suitable for tires in the automobile industry. From elemental analysis, the presence of nitrogen was detected for the collagen-filled SBR composite, proving the successful incorporation of collagen fibrils. The physicochemical analysis also detected a trace of graphene oxide and collagen functional groups in the SBR composite. In addition, the thermal analysis revealed those collagen-filled composites had stable heat tolerance behavior, which is suitably used in extreme weather conditions. Moreover, the SBR/GO/1.5COL sample exhibited good characteristics in both mechanical and biodegradable properties. Thus, the product of SBR/GO/1.5COL could be regarded as a promising composite for green tires in the auto industry in the future.

## 1. Introduction

The utilization of a bio-based green composite to make automobile parts using renewable resources has become a common industrial practice [1]. For decades, the abandoned rubber tires have been in great distress or annoyance for environmentalists and landfills. According to a recent study, around 5.1 billion tires are abandoned all around the world very year. The toughness of the old tires is the biggest obstacle after they have been landfilled and covered up by garbage, which bears inestimable time for decomposition to take place. Adversely, if not covered, it will collect rainwater and cause the growth of insect-like mosquitoes that are harmful to humans. Despite the decomposition in due course, it still contains some dangerous chemicals leaching into the soils that might pose a risk to the environment. Currently, used tires are recycled to make shoes, bushings, washers, gaskets, wheels, compartments, and while a wide variety of items for the household, business, and modern application [2]; however, it is not a true solution for the long term.

The main constituent of tires is rubber, and over 70% of the world’s rubber is created in just three nations (Thailand, Indonesia, and Malaysia), and around 40% was obtained by just three nations (United States, China, and Japan). The vast majority of the common rubber (75%) is utilized for the creation of vehicle tires [3]. Both natural rubber and styrene-butadiene rubber (SBR) make up 60% of the world’s rubber market, and their fabrication process on a large scale is environment friendly making it preferably selected as a matrix material in the tire industry. The consumption of rubber tire waste produces a lot of warmth and smoke, which is one of the primary variables for a worldwide temperature alteration [4].

Scientists around the globe are concerned about waste tires and are working to find a highly efficient, biodegradable material that can replace non-biodegradable rubber by modifying natural and synthetic rubber by functionalizing with different moieties or by using fillers, which could be more efficient, environmentally friendly, biodegradable, and biocompatible as compared to the natural or synthetic rubber [5]. However, this always remains a challenging subject of biodegradable tire material for environmental health. According to the literature, many fillers can be used for the improvement of SBR quality, such as metals, metal oxides, silica, graphite, carbon black, carbon nanotubes, graphene oxide, and graphene. However, graphene oxide and graphene generally showed the best results.

Graphene oxide [6,7,8], having unique physical properties, emerged as a new nano-sized and layered material for rubber reinforcement [9,10,11]. The precursor of graphene oxide is naturally abundant and oil independent. Current studies of graphene oxide and graphene are concerned with thermal and functional properties, but in the tire industry, the mechanical, biodegradable, and environmental issues are of more concern [12,13,14,15]. Moreover, graphene nanosheets are idle in the tire industry by attributing to their unique 2D geometric architecture and unique aspect ratio, besides their great mechanical, thermal, optoelectronic, and chemical stability properties. Despite all these properties providing it with great potential in bionanotechnology, it remains in limited use due to the lack of biocompatibility, low aqueous solubility, featureless surfaces, and highly hygroscopic characteristics [15]. To overcome these disadvantages, some biomolecules could be functionalized on the graphene surface to improve their properties [16].

Amyloid fibril protein composites with graphene sheet is an excellent idea for enhancing biocompatibility and biodegradability. Hybride collagen–apatite platelets in bone have a strong laminated structure with a natural composition. Meanwhile, hybrid cellulose-based graphene nanocomposites have also been reported [4,16]. Thus, it is promising to apply these ideas to prepare rubber composite by replacing apatite platelets with graphene nanosheet and amyloid fibrils with collagen fibrils. This composite is potential for the automobile tire industry and would contribute enormous opportunities for preparing high-performance rubber composites in future engineering applications.

Collagen is one of the most abundant proteins in the extracellular matrix of mammals. It is found in connective tissues and plays a vital role in developing skin, bones, and tendons [17]. Due to their biodegradability and biocompatibility, collagen-based biomaterials are used in tissue engineering, mainly for applications in wound-healing and biomineralization [17]. The main goal of tissue regeneration is to reestablish the fragile network of collagen fibrils that allows for proper physiological recovery. Collagen, unlike other proteins, has a unique structure made up of three left-handed polyproline-II=type polypeptide chains coiled to create a triple-helical shape. The self-assembly of collagen molecules or triple helices into fibrils would generate tissue structure biological activities for cellular functions and applications [17]. The strength and mechanical flexibility of collagen-based materials are important factors in material engineering. Collagen is mainly extracted from mammalian sources, especially bovine and porcine, for cosmetic and biomedical uses. However, these collagen sources are not widely accepted due to contamination risk of diseases, such as bovine spongiform encephalopathy and transmissible spongiform encephalopathy [18]. As a result, the demand for collagen from alternative sources, especially fish byproducts, has increased in recent years [19]. Collagen can be extracted by treating with neutral salt, acidic, and acid solutions containing enzymes. In traditional extraction and production of collagen-rich products, including partially degraded and denatured collagen (i.e., gelatin), the collagen-rich parts of the animals, like the skin, hoofs, tendons, bones, and cartilage, are separated and processed. Separating collagen-rich parts from larger animals such as bovine and porcine is relatively easy and is done manually in the industry [20].

Concerning the structural, thermal, and mechanical properties of rubber for the tire industry fulfilled by graphene, its biodegradability would be achieved by increasing its biocompatibility. Hence, in this research study, we aim to produce SBR rubber composite with graphene oxide and make it biodegradable by adding a biomolecule filler. For that, we would incorporate biodegradable graphene into SBR by functionalizing the graphene with degradable elastin collagen biomolecule. The prepared SBR composite would be characterized by various techniques analysis such as Field-Emission Electron Microscopy (FESEM), Energy Dispersive X-ray (EDX), Brunauer–Emmett–Teller (BET), Thermogravimetry Analysis (TGA), Differential Scanning Calorimetry (DSC), Fourier Transform Infrared spectroscopy (FTIR), and mechanical tensile tests to understand the physicochemical properties of this composite. Meanwhile, the biodegradability of SBR composites was evaluated to examine their suitability for green tire applications, which would open up gigantic chances for future development of tire composite products. The produced SBR composite tires in this work would be regarded as ‘green tires’, which have the biodegradable property and decomposition action after landfilling in soil.

## 2. Materials and Methods

### 2.1. Materials and Chemicals

SBR rubber granules (Mooney viscosity: 47 MU; Bound styrene: 23.6%) was procured from Zibo Feitian International Trading Co., Ltd. (Shandong, China). Other materials like nanographene oxide, elastin collagen granules (Alpha chain: Type I; Chemical structure: C_57_H_91_N_19_O_16_; Molecular weight: 300 kDa), butadiene-styrene-vinyl-pyridine rubber (VPR) latex, sulfuric acid (H_2_SO_4_) (95–98%), hydrazine (N_2_H_4_), phosphorus pentoxide (P_2_O_5_), and dialysis tube (10 mm average flat width, 2000 MWCO) were purchased from Sigma-Aldrich Sdn. Bhd., Malaysia. All chemicals were research-grade and used as received.

### 2.2. Preparation of Composites

Exfoliated nanographene oxide (GO) was used as the source for graphene. The GO could be converted into coated stable monolayer dispersions with the presence of elastin collagen (COL) fibrils in a pH range of 2–8, while the GO precursors have a negative static charge. The preparation of GO solution and SBR composites was conducted according to the reported literature works with some modifications [21,22].

To prepare GO solution, about 1.0 g GO was dispersed in 100 mL of water, maintained at pH 2 under 2 h continuous sonication. After that, the dispersion was centrifuged at 3000 rpm. for 30 min to remove insoluble aggregates. The supernatant was collected and dialyzed with Millipore water at 48 °C for 7 days until reaching pH 2 value to remove any traces of salts and acids.

A pure SBR sample was prepared by melting 2.0 g of SBR granules in 200 mL of a chloroform solution. It was then evaporated at room temperature for 24 h, followed by oven-drying with P_2_O_5_ at 40 °C overnight before compression molding to form a solid rubber structure. For preparing an SBR/GO and SBR/GO/COL composite, a collagen solution (containing 1.5 wt% or 2.5 wt% amounts) was mixed with GO solution under vigorous stirring. The final volume of the solution was 50 mL, and the weight ratio of the GO was 5.0 wt% since this amount could provide stabilized structure for SBR rubber after we conducted the trial and fail work for SBR using different GO amounts to obtain optimized results. Afterward, the GO was partially reduced to graphene by adding 50 mL N_2_H_4_ reducing agent into the solution with constant stirring, maintained at 60 °C for 20 h to generate a well-assimilated nano-sheet structure after being mixed with SBR in later steps. The mixture was kept at 48 °C after cooling. Hereafter, SBR rubber granules with a 6-mm diameter were mixed and vigorously stirred for 30 min.

About 1.0 vol% of H_2_SO_4_ and 2.0 vol% VPR latex were then added for coagulation to obtain the emulsion of SBR/GO/COL. Finally, the obtained coagulated composite was washed with Millipore water until pH 6–7 to remove any free form of acid and salt, then followed by oven-drying with P_2_O_5_ at 40 °C for overnight. The composite was further subjected for compression by using a standard mold at an optimal applied pressure of 15 MPa and temperature of 150 °C as determined with the help of a disc rheometer. Each composite sample with a changing ratio of graphene, collagen fibril, and SBR would be prepared according to the designed formulation, as shown in Table 1.

### 2.3. Characterization

#### 2.3.1. Morphology

The surface morphology of the SBR composites was investigated by a field emission electron microscope (FESEM) under an accelerating voltage in the range of 10–20 kV. Before FESEM observation, the composites were coated with platinum on a carbon-taped stub via sputtering to avoid the charging effect.

#### 2.3.2. Porosity

Brunauer–Emmett–Teller (BET) analysis was conducted to determine the composite porosity and average pore size by employing a Micromeritics ASAP 2020 PLUS instrument, which is specialized and can be used for testing film samples. The film samples were cut down to a 0.5 × 0.5 cm^2^ dimension size with around 0.15–0.20 g sample mass before degassing in the flow of nitrogen gas at 50 °C for 30 min. After degassing, the samples were subjected to nitrogen gas absorption at a 77 K bath temperature with 10 s equilibrium interval time for porosity analysis.

#### 2.3.3. Chemical Functionality

The chemical functionality of the composite samples was examined with a Perkin Elmer 1600 infrared spectrometer (FTIR) in 500–4000 cm^−1^ wavenumber range at 4 cm^−1^ resolution. The positions of significant transmittance peaks at a particular wavenumber were tracked using Nicolet software version 7.3.

#### 2.3.4. Thermal Property

The changes in thermal stability of the composite samples were examined with a TA Instruments Q500 thermogravimetric analyzer (TGA) in a temperature range of 25–1000 °C at a 20 °C/min heating rate. Meanwhile, a TA Instruments Q20 differential scanning calorimeter (DSC) was also performed to study the thermal behavior changes of composite in a temperature scanning range from 25 °C to 300 °C at 20 °C min^−1^ heating rate under nitrogen atmosphere.

#### 2.3.5. Mechanical Property

An Instron 4400 Universal Tester was used to study the mechanical properties of composites with respect to tensile strength and elongation at break of the samples according to the D412 (standard tensile test for rubber) under a fixed 12.5 mm/min crosshead speed. The composite samples were cut into 20 mm × 60 mm sizes before analysis with a triplicate run.

#### 2.3.6. Biodegradability

The biodegradability of the composite samples was studied according to their weight loss changes after being landfilled in soil for 50 days. During preparation, the composite samples were cut into 20 mm × 60 mm size and then embedded in 0.5-m-deep soil.

## 3. Results and Discussion

### 3.1. Morphology, Porosity, and Elemental Composition

**Figure 1:**
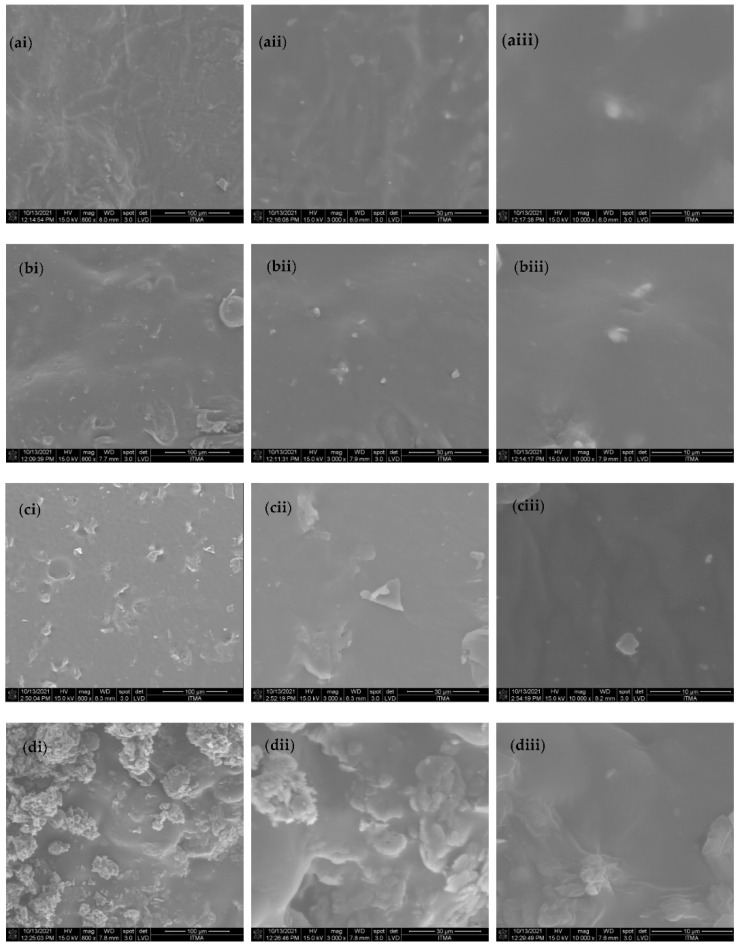
FESEM image of (**a**) pure SBR, (**b**) SBR/GO, (**c**) SBR/GO/1.5COL, and (**d**) SBR/GO/2.5COL under (**i**) 800×, (**ii**) 3000×, and (**iii**) 10,000× magnifications.

Figure 1 displays the FESEM image of SBR samples mixed with different amounts of graphene and collagen fillers. From Figure 1a, the sample of pure SBR had flat surface morphology. Meanwhile, it also presented wrinkle-like and protuberant features. This was because of the asymmetrical interaction between SBR polymeric chains, which was caused by the repulsion effect of π orbital and double bonding that existed in SBR rubber [16]. When mixed with graphene oxide, the morphology had slightly changed for SBR/GO sample by showing prominent ridges surface (Figure 1b). However, the apparent feature had become compact and well-assimilated, indicating the incorporation of graphene nanosheet could improve the structure of SBR polymer [23]. Apart from that, there were more protuberant and pit features observed for SBR/GO/1.5COL sample (Figure 1c). This implied the 1.5 wt% collagen filler was successfully embedded inside the GO-exfoliated SBR polymer matrix. Moreover, in Figure 1d, numerous clumps of the aggregated structure had appeared for SBR/GO/2.5COL sample, showcasing the increased addition of collagen to 2.5 wt% could not interact well with SBR polymer [24].

Figure S1 illustrated the EDX spectra of SBR samples, while their elemental composition was tabulated in Table 2. The pure SBR sample revealed the carbon atom as its major compositional element, indicating it possessed the typical characteristic of SBR molecules. Besides this, the SBR/GO sample showed reduced in carbon atom element, whereas with increased oxygen atom content, proving the successful incorporation of graphene oxide sheet [13]. Meanwhile, the carbon amount was further decreased in SBR/GO/1.5COL sample, along with the increased oxygen content and presence of nitrogen element, which attributed to the proteinous content of collagen fibrils. As for the SBR/GO/2.5COL sample, it showed a significant reduction of carbon and increment of oxygen but presented only a slight increment of nitrogen when compared to SBR/GO/1.5COL. It was due to the extremely small proportion of collagen fibrils built up in the composite despite the great contain 2.5 wt% filing amount [16].

From Table 2, the pure SBR sample revealed the lowest surface area among the samples, implying its relatively flat surface feature. However, it possessed the highest pore volume and pore size compared to other samples, which formed by the repelling effect between the SBR molecular chains in the existence of double π-bonds. Apart from that, the porosity was gradually improved for the SBR/GO sample with reduced pore volume and pore size after the incorporation of the nanographene oxide sheet. This indicated that more integrated morphology was formed to the SBR/GO sample. The increased surface area was likely due to the asymmetrical surface feature of the SBR/GO sample [12]. The increment of the surface area was more prominently observed for the SBR/GO/1.5COL sample owing to the formation of pits and protuberant structure. However, the porous size and volume were not affected much to SBR/GO/1.5COL, showcasing good interaction between the composite components following the addition of 1.5 wt% collagen fibrils [11]. By increasing to 2.5 wt% collagen amount, all features, including the surface area, pore size, and pore volume, were greatly affected for SBR/GO/2.5COL sample. It was resulted by agglomeration of collagen fibrils as aforementioned in FESEM analysis.

### 3.2. Thermal Stability

**Figure 2:**
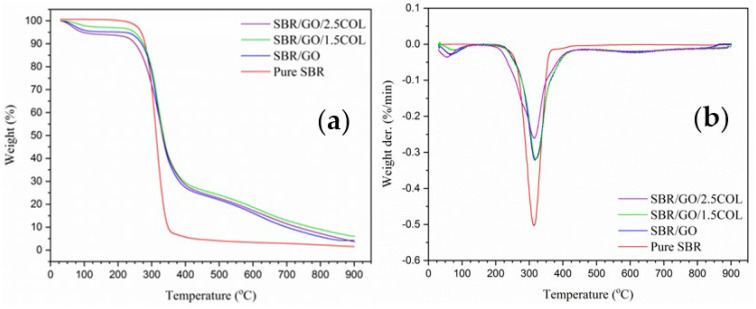
TGA (**a**) and DTG (**b**) curves of SBR samples.

TGA curves of SBR samples are presented in Figure 2a. Both SBR/GO/1.5COL and SBR/GO/2.5COL samples showed initial decomposition temperature at 281.9 °C and 279.5 °C, respectively, which higher than the samples of SBR/GO at 261.6 °C and pure SBR at 276.5 °C. It was probably due to the good componential interaction in collagen-filled composite, contributing to the stable thermal degradation behavior with improved heat resistance [16]. Besides this, both collagen-filled composites also showed uniform weight loss behavior as same as SBR/GO sample, indicating their bio-components interacted well with each other [15]. From DTG curves (Figure 2b), SBR/GO/1.5COL and SBR/GO/2.5COL showed higher peak decomposition temperature at 320.7 °C and 318.6 °C respectively, compared to SBR/GO at 315.7 °C and pure SBR at 316.0 °C. These thermal results proved that the fabricated collagen-filled SBR composite could be used as tire material to resist the high temperatures of possible applications in the automobile industry.

**Figure 3:**
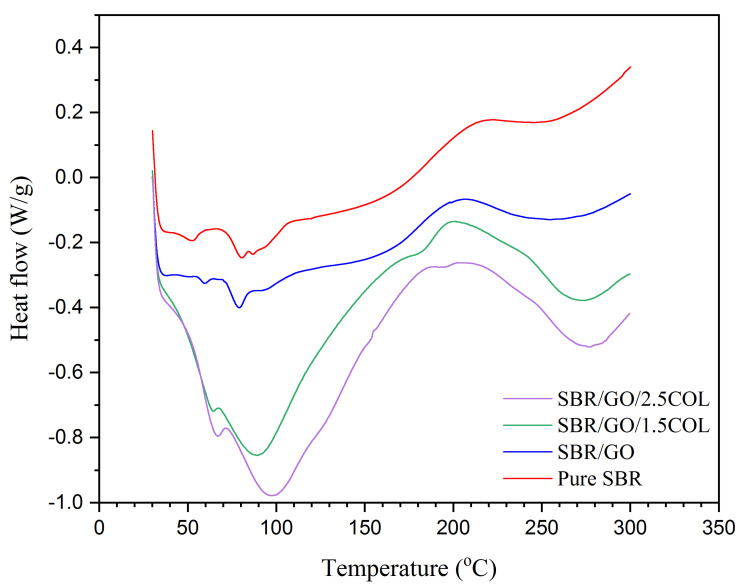
DSC spectra of SBR samples.

Figure 3 shows the DSC spectra of SBR samples. The crystallization temperature was revealed as the first endothermic band at 52.8 °C for pure SBR. However, its melting temperatures were observed in two endothermic bands at 80.3 °C and 86.8 °C, indicating its melting behavior was inconsistent and non-uniform. After nanographene incorporation, the thermal behavior was not much different for SBR/GO when compared with pure SBR samples. The crystallization temperature was enhanced to 59.4 °C for SBR/GO, while its melting temperature was rather presented as a single endothermic band at 79.2 °C with a shoulder band observed at 92.4 °C. This showcased the thermo-molecular change in SBR/GO is mildly more stable than pure SBR [24]. With the addition of collagen fibrils, the thermal property became unstable for SBR/GO/1.5COL and SBR/GO/2.5COL samples by showing broader endothermic bands from 40 °C to 160 °C, owing to the absorption of heat energy to break down the collagen protein interactions for denaturation as well as for water evaporation process. The crystallization temperature was slightly improved to 63.9 °C and 66.2 °C for both SBR/GO/1.5COL and SBR/GO/2.5COL samples, respectively. Meanwhile, their melting temperature was largely enhanced to 88.9 °C and 97.7 °C temperatures, respectively, which was promoted by the colloidal effect of collagen fibrils. Between 200 °C to 250 °C, heat absorption had occurred, which was readily used for decomposition of SBR rubber that occurred beyond 250 °C temperature [23].

### 3.3. FTIR

**Figure 4:**
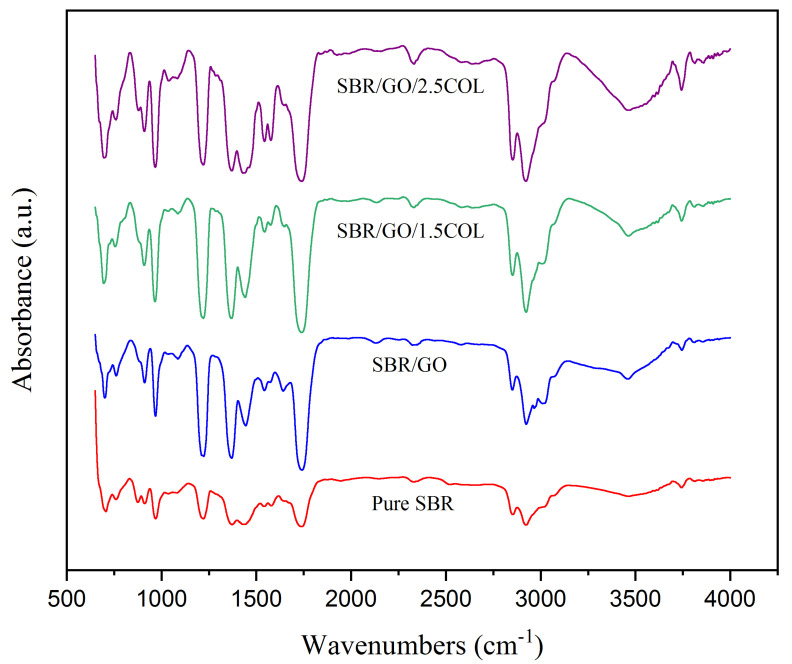
FTIR spectra of SBR samples.

FTIR spectra of SBR samples are presented in Figure 4. The pure SBR sample showed significant peaks at 2851 cm^−1^ and 2922 cm^−1^, which corresponded to the C-H stretching of SBR molecular chains. Meanwhile, another prominent peak noted at 1732 cm^−1^ was in response to the C=C double bonds vibration in SBR aromatic ring. In addition, the peaks at 1219 cm^−1^, 1371 cm^−1^, and 1438 cm^−1^ were assigned to the C-C bending of the SBR backbone structure. Moreover, the 704 cm^−1^ peaks were attributed to the CH_2_ rocking vibration, and the 968 cm^−1^ peaks were related to the =C-H bending vibration of SBR [12]. With the incorporation of graphene oxide, those SBR characteristic peaks intensities had changed gradually due to the presence of carbonic sigma bonding structure in the SBR/GO sample. In addition, the O-H group vibration had appeared at 3452 cm^−1^, while the peak intensities at 1219 cm^−1^ (C-O stretching) and 1732 cm^−1^ (C=O stretching) relating to the COOH group had increased significantly for SBR/GO sample. Besides this, the peak intensities were noted further increasing at 3452 cm^−1^ (O-H vibrate), 2922 cm^−1^ (C-H stretch), 2851 cm^−1^ (C-H stretch), 968 cm^−1^ (=C-H bend), and 704 cm^−1^ (CH_2_ vibrate) for SBR/GO/1.5COL and SBR/GO/2.5COL samples, which promoted by the protein characteristic of collagen fibrils [14]. Additionally, a tremendous increment of peak intensity was noticed for SBR/GO/2.5COL sample at 1575 cm^−1^ (C=C aromatic vibration), 1542 cm^−1^ (C=O vibration), and 1440 cm^−1^ (C-N bending), that driven by the increased collagen amount [13].

### 3.4. Mechanical and Biodegradability Test

**Figure 5:**
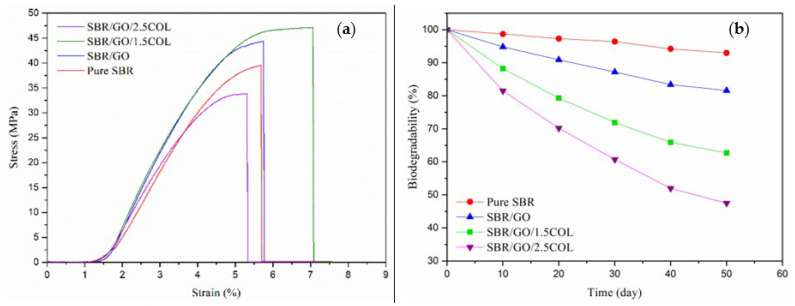
Mechanical stress–strain (**a**) and biodegradability (**b**) tests of SBR samples.

Figure 5a presented the mechanical stress–strain test of SBR samples, and the analyzed data were listed in Table 3. The SBR/GO sample showed greatly enhanced tensile strength while with slightly improved elongation at break, implying better polymeric interaction compared to pure SBR [6]. Meanwhile, both tensile strength and elongation at break were improved for SBR/GO/1.5COL composite sample. This was attributed to the great interface and adherence between biocomponents that imparted the property of effective stress transfer. However, SBR/GO/2.5COL presented lower elongation at break and tensile strength than other composite samples. This was possible because the aggregated collagen fibrils resulted in the decreased stress–strain transfer in the polymer matrix [9]. In terms of Young’s modulus, the SBR/GO/1.5COL composite gave the highest value of 1.51 GPa, whereas SBR/GO/2.5COL composite displayed the lowest value of 1.32 GPa. Hence, the mechanical test results suggested the SBR/GO/1.5COL composite had the greatest elasticity, stiffness, and rigidity among the samples.

For the biodegradability test (Figure 5b), the sample of pure SBR showed almost unchanged weight loss after being landfilled for 50 days in soil due to its non-degradable feature. However, the biodegradability had been improved for the SBR/GO sample by showing more than 10% weight loss but remained with nearly 85% constant weight after 50 days. This was due to the introduced graphene oxide possessing a naturally-bonded carbon structure [13]. Additionally, the biodegradability was greatly enhanced to more than 30% and 50% for SBR/GO/1.5COL and SBR/GO/2.5COL samples, respectively, owing to the biodegradable nature of collagen. Thus, SBR/GO/1.5COL was a promising composite for acting as a green tire in the automobile industry by attributing to its good in both mechanical and biodegradable properties. Such high biodegradability is required for tires since it would allow the decomposition process to take place once embedded in the soil. The biodegradation process of the tire will only be initiated after landfilling due to the presence of microbial and certain chemical reactions that occur in the soil [15]. When applied for use in automobiles, biodegradation would be slow and unlikely to happen. In this work, both GO and COL components are regarded as bio-materials, which could promote the biodegradation property of SBR rubber. The GO was functionalized with COL with the aim to further enhance its biodegradability. Hence, both GO and COL components played synergistic roles in enhancing the biodegradability of SBR rubber.

## 4. Conclusions

The present work revealed the major findings of the successful incorporation of biodegradable collagen-functionalized nanographene oxide sheets into an SBR rubber composite. From the microscopy investigation, the morphology of pure SBR improved after the addition of GO for SBR/GO composite by revealing a compact structure with a smoother surface. As for the SBR/GO/1.5COL sample, the COL filler was found effectively embedded in the SBR/GO matrix, proving the 1.5 wt% COL filler amount could be incorporated into the composite with good interfacial interaction. However, when the amount increased to 2.5 wt% COL, it led to the formation of an aggregated structure in the SBR/GO/2.5COL sample due to an unreacted interface between COL filler and SBR/GO. Hence, the produced SBR composite in this work can be utilized for future green tire applications in the automobile industry.

## Figures and Tables

**Table 1:** Denotations and formulation of prepared SBR composite.

Samples of Composite	Formulation		
	SBR (wt%)	GO (wt%)	COL (wt%)
SBR	100.0	-	-
SBR/GO	95.0	5.0	-
SBR/GO/1.5COL	93.5	5.0	1.5
SBR/GO/2.5COL	92.5	5.0	2.5

**Table 2:** Elemental composition, surface area, and porous structure analysis data of SBR samples.

Samples	Carbon (%) ^a^	Oxygen (%) ^a^	Nitrogen (%) ^a^	Surface Area (m^2^/g) ^b^	Pore Volume (cm^3^/g) ^b^	Pore Size (nm) ^b^
Pure SBR	100.0	-	-	2.76	0.345	249.75
SBR/GO	92.73	7.27	-	3.91	0.168	85.83
SBR/GO/1.5COL	91.40	8.04	0.56	4.51	0.186	82.68
SBR/GO/2.5COL	87.70	11.71	0.58	4.10	0.325	158.49

^a^ EDX analysis; ^b^ BET analysis.

**Table 3:** Mechanical test data of SBR samples.

Membranes	Tensile Strength (MPa)	Elongation at Break (%)	Young’s Modulus (GPa)
Pure SBR	39.4	5.8	1.34
SBR/GO	44.2	5.9	1.46
SBR/GO/1.5COL	47.0	7.2	1.51
SBR/GO/2.5COL	33.8	5.4	1.32

## Data Availability

Not applicable.

## Supplementary Materials

The following supporting information can be downloaded at: https://www.mdpi.com/article/10.3390/gels8030161/s1. Figure S1. EDX spectra of (a) pure SBR, (b) SBR/GO, (c) SBR/GO/1.5COL, and (d) SBR/GO/2.5COL samples.
