# Supplementary figures and images for: Preparation of Styrene-Butadiene Rubber (SBR) Composite Incorporated with Collagen-Functionalized Graphene Oxide for Green Tire Application
Source: Gels. 2022 Mar 4;8(3):161. doi: 10.3390/gels8030161 (PMC8951021; doi:10.3390/gels8030161)

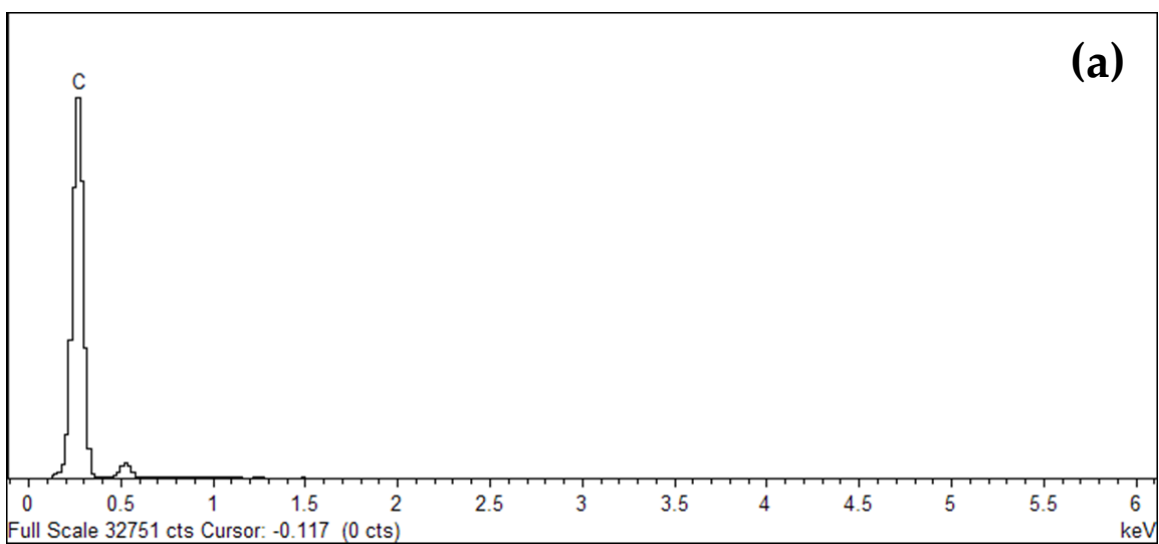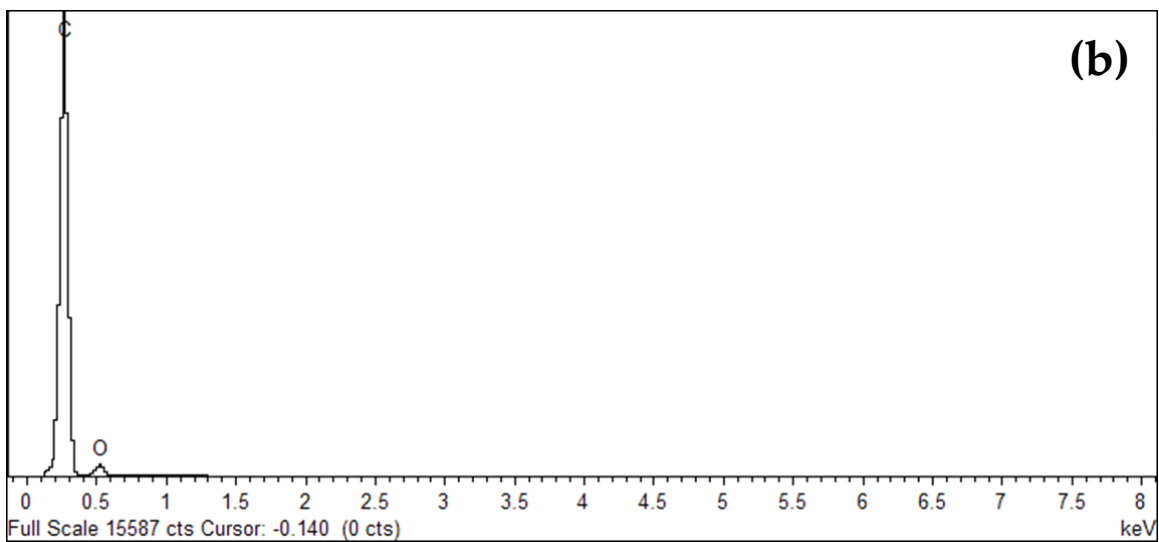

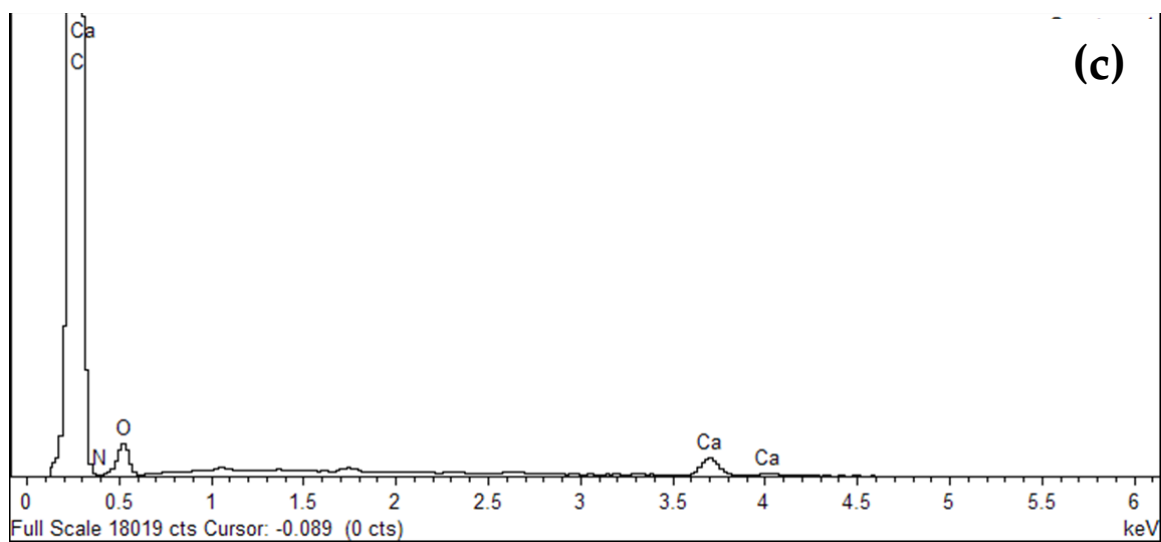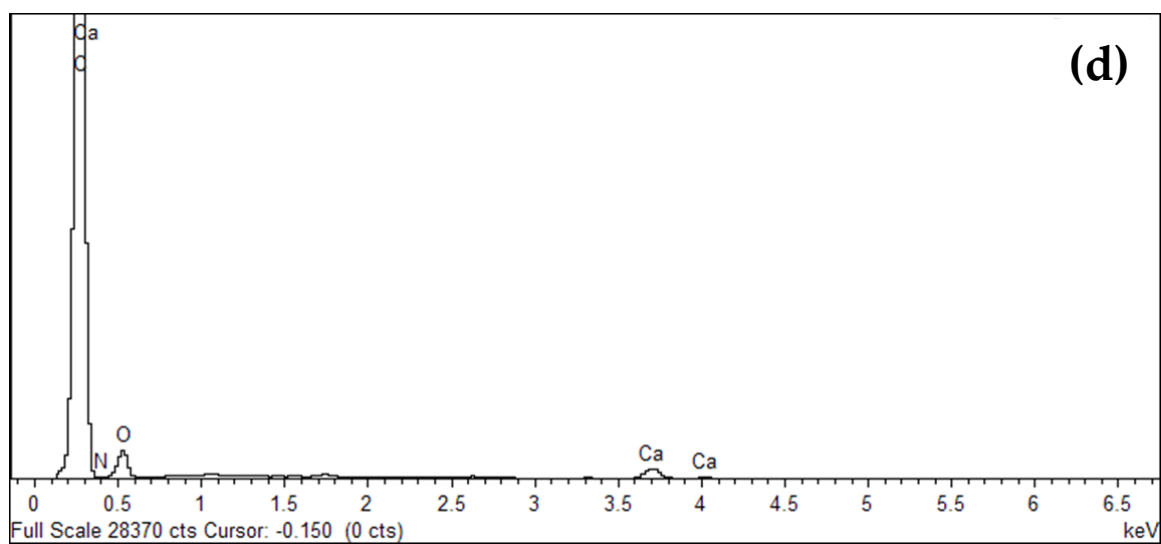

**Figure S1.** EDX spectra of (a) pure SBR, (b) SBR/GO, (c) SBR/GO/1.5COL, and (d) SBR/GO/2.5COL samples.

Supplement: Supplementary file 1 [file gels-08-00161-s001.zip › gels-1584502-supplementary.pdf]
